# *HER2/neu* overexpression in the development of muscle-invasive transitional cell carcinoma of the bladder

**DOI:** 10.1038/sj.bjc.6601245

**Published:** 2003-09-30

**Authors:** Z Latif, A D Watters, I Dunn, K M Grigor, M A Underwood, J M S Bartlett

**Affiliations:** 1 University Department of Surgery, Level II, Queen Elizabeth Building, Glasgow Royal Infirmary, Glasgow G31 2ER, UK; 2 University Department of Pathology, Western General Hospital, Edinburgh EH4 2XU, UK

**Keywords:** transitional cell carcinoma, oncogenes, *HER2/neu*

## Abstract

The mortality from transitional cell carcinoma (TCC) of the urinary bladder increases significantly with the progression of superficial or locally invasive disease (pTa/pT1) to detrusor muscle-invasive disease (pT2+). The most common prognostic markers in clinical use are tumour stage and grade, which are subject to considerable intra- and interobserver variation. Polysomy 17 and *HER2/neu* gene amplification and protein overexpression have been associated with more advanced disease. Standardised techniques of fluorescence *in situ* hybridisation and immunohistochemistry, which are currently applied to other cancers with a view to offering anti-*HER2/neu* therapies, were applied to tumour pairs comprising pre- and postinvasive disease from 25 patients undergoing treatment for bladder cancer. In the preinvasive tumours, increased *HER2/neu* copy number was observed in 76% of cases and increased chromosome 17 copy number in 88% of cases, and in the postinvasive group these values were 92 and 96%, respectively (not significantly different *P*=0.09 and 0.07, respectively). *HER2* gene amplification rates were 8% in both groups. Protein overexpression rates were 76 and 52%, respectively, in the pre- and postinvasive groups (*P*=0.06). These results suggest that *HER2/neu* abnormalities occur prior to and persist with the onset of muscle-invasive disease. Gene amplification is uncommon and other molecular mechanisms must account for the high rates of protein overexpression. Anti-*HER2/neu* therapy might be of use in the treatment of TCC.

Transitional cell carcinoma (TCC) of the urinary bladder is common in the UK, with over 15 000 new cases being diagnosed annually. The low mortality from superficial disease contributes to this being the second most prevalent cancer in the UK population. However, with the development of detrusor muscle invasion, mortality rates increase significantly, and over 50% of patients already have micrometastasis on diagnosis of detrusor muscle-invasive disease. Therefore, more aggressive clinical treatment needs to be applied, if there is a curative aim, in the form of radical surgery or radiotherapy plus adjuvant treatment (Skinner *et al*, 1991). Approximately 10–15% of superficial or locally invasive (pTa/pT1) tumours progress to muscle invasion, and this risk is dependent on tumour stage and grade. For example, well-differentiated (grade 1) tumours progress in only 2% of cases, whereas poorly differentiated (grade 3) tumours progress in up to 20% of cases. However, stage and grade are subject to 50% inter- and intraobserver variation (van der Meijden *et al*, 2000). Therefore, more accurate prognostic factors are desirable, and genetic markers might fulfil this role (Reznikoff *et al*, 2000).

Polysomy 17 in TCC is a chromosome-specific event, independent of tumour polyploidy (Bartlett *et al*, 1999) and is associated with higher tumour stages and grades as well as disease recurrence, progression and decreased patient survival (Bartlett *et al*, 1998; Li *et al*, 1998; Watters *et al*, 2000). Polysomy 17 has been observed in 10% of grade 1 and 43% of grade 3 tumours (Watters *et al*, 2000) and 32% of pTa/T1 and 72% of pT2 tumours (Li *et al*, 1998). Gain of chromosome 17 may therefore be a useful marker of tumour progression.

The *HER2/neu* oncogene is located on chromosome 17 q11-21 and encodes for a tyrosine kinase *trans*-membrane growth factor receptor. Activation of the *HER2/neu* receptor, following autophosphorylation of the tyrosine kinase residues results in the activation of a cascade of intracellular proteins. Ultimately, the mitotic activity and metastatic potential of the cell increases (Underwood *et al*, 1995; Tzahar *et al*, 1996; Olayioye *et al*, 2000).

*HER2/neu* protein overexpression, assessed by immunohistochemistry (IHC), has been associated with increased tumour grade in TCC, but there is a wide variation in the literature of 2–50% (Wright *et al*, 1990; Coombs *et al*, 1991; McCann *et al*, 1990; Underwood *et al*, 1995; Mellon *et al*, 1996). This may be due to the application of different antibodies and scoring systems. Similarly, although *HER2/neu* gene amplification rates are higher in muscle-invasive disease compared with superficial disease, there is a wide variation in the literature of 4–32% (Coombs *et al*, 1991; Underwood *et al*, 1995; Mellon *et al*, 1996). Accurate assessment of the prognostic significance of HER2 in the progression of bladder cancer requires standardisation of the laboratory techniques. Current UK guidelines recommend the use of IHC, with the use of specific antibodies and scoring systems to assess protein overexpression, and fluorescence *in situ* hybridisation (FISH) to assess gene amplification. Our centre is one of the designated centres in the UK that has the facilities to apply such techniques (Ellis *et al*, 2000). This is particularly important if the *HER2/neu* oncogene is to be targeted with one of the various anti-*HER2/neu* therapies being used in the treatment of other cancers. In breast cancer, tumours that have evidence of *HER2/neu* gene amplification, or strong protein overexpression respond to treatment with the anti-*HER2/neu* monoclonal antibody Trastuzumab (Herceptin, Gnenetech Inc San Francisco, USA). Response rates of 50% have been observed in combination with chemotherapy and 26% as monotherapy in women with metastatic breast cancer as well as an increased time to progression (Slamon *et al*, 2001; Vogel *et al*, 2001).

The aim of the present study was to assess *HER2/neu* protein overexpression and gene amplification in 25 tumour pairs. The first tumour of the pair was pre (muscle) invasive and the second tumour (from the same patient) was postinvasive.

## MATERIALS AND METHODS

### Patients

Patients with tumours that had progressed from superficial disease (pTa/pT1) to muscle-invasive disease (pT2) were identified from a bladder cancer database in the Department of Surgery, Glasgow Royal Infirmary. In order to assess HER2 abnormalities during disease progression to muscle invasive disease, pTa/pT1 and pT2 tumours from the same patient were compared. All patients had full clinical follow-up (age, date of diagnosis, cystoscopic follow-up, tumour stage and grade and survival). Ethical approval was obtained for these studies. (5 *μ*g) Sections of formalin-fixed paraffin tissue were cut onto sialinised slides and baked at 56°C overnight. All representative TCCs analysed had one section stained with haematoxylin and eosin (H&E), and were restaged and regraded by a specialist urological pathologist (KMG). The pathologist rejected 52 tumours initially selected for the study because of the absence of detrusor muscle in either the pre- or postinvasive tumour. In order to be accepted for the study, both pre- and postinvasive tumours had to have detrusor muscle in both specimens.

### Fluorescence *in situ* hybridisation

The FISH methodology was followed as outlined: tissue sections were dewaxed and rehydrated, then subject to pretreatments with 0.2 N HCL for 20 min at room temperature, 8% sodium thiosulphate at 80°C for 30 min, and 0.5% pepsin in 0.01 N HCL for 26 min at 37°C. Tissue sections were postfixed in 10% neutral buffered formalin at room temperature for 10 min before dehydration in ascending grades of alcohol and air drying. These steps were carried out on a VP2000 robotic pretreatment slide processor (Vysis, UK, Ltd). The tissue sections were assessed for the extent of tissue digestion (Watters *et al*, 2000). Tissue sections were denatured in 70% formamide, 2 × SSC, pH 7–8 at 72°C for 5 min on the Omnislide hybridisation module (Hybaid, UK, Ltd). Probes for the pericentromeric region of chromosome 17 (SpectrumGreen™) and the locus specific probe for HER2 (SpectrumOrange™) were used. For each section, 1 *μ*l of each probe was added to 7 *μ*l hybridisation mix (50% formamide, 2 × SSC, 10% dextran sulphate) and 1 *μ*l deionised water and denatured in a water bath at 72°C for 5 min and then hybridised overnight at 37°C. Posthybridisation washes were in 0.4 × SSC, Nonidet 30, pH 7, at 72°C for 2 min. The sections were mounted in 0.25 *μ*g ml^−1^ DAPI antifade (Veactashield, UK) and viewed with a Leica DMLB microscope. A triple band pass filter block spanning the excitation and emission wavelengths of the SpectrumOrange™ and SpectrumGreen™ and DAPI was used in the analysis of the hybridisation. Image capture was achieved using a digital camera (Leica DC 200, Leica, UK).

### Fluorescence *in situ* hybridisation scoring

Serially sectioned haemtoxylin- and eosin-stained tissue sections were first examined to localise areas of TCC. Fluorescence *in situ* hybridisation sections were then scanned at × 400 magnification to localise the areas of interest. In total, three areas were identified and in each area 20 nuclei were assessed. Chromosome 17 copy number and HER2 copy number were assessed for each of the 20 nuclei at × 1000 magnification. An average chromosome 17 copy number and *HER2/neu* copy number was obtained totalling the number of signals over the 60 nuclei and dividing by the number of signals. Control sections of normal bladder and *HER2/neu* gene amplified breast tumours were included in each run. The values for disomy were derived from the analysis of normal bladder post-mortem tissue, as previously assessed (Bartlett *et al*, 1998; Watters *et al*, 2000). The average *HER2/neu* copy number was 1.7 (±0.1) and hence a *HER2/neu* copy number greater than 2 (1.7+3 × s.d.) was defined as ‘increased’. The average chromosome 17 copy number was 1.7 (±0.06) and hence a polysomy 17 was defined as a chromosome 17 copy number greater than 1.88 (1.7+3 × s.d.). Gene amplification was defined as an *HER2*/Chromosome 17 ratio of greater than 2 (Bartlett, 2001), based on the value used in breast cancer diagnostics.

### Immunohistochemistry

Antigen retrieval was performed by placing the slides in a pressure cooker containing 1 l of boiling water with 0.37 g (1 × 10–3 moles) of ethylenediaminetetracetic acid (EDTA). The pressure cooker was placed in a microwave (850 W) for 13.5 min, then the lid was removed and the slides were left to stand for another 20 min. The slides were then loaded onto an automated machine (NEXUS II, Ventana USA) with a rotating slide carousel. The following reagents were added in sequence automatically by the machine (all steps were performed at 37°C, and reagents were purchased prepacked): (1) 0.1 ml of inhibitor (containing 1.1% hydrogen peroxide, which is metabolised by endogenous peroxidase), for 4 min; (2) 100 *μ*l (0.63 g ml^−1^) of CB11 monoclonal primary antibody (IgG 1); (3) 0.1 ml each of amplifier A (IgG heavy and light chains) and B (IgG heavy chains) for 8 min. This binds to the previously bound primary antibody, increasing the number of antibodies at the site of the antigen, thereby increasing staining intensity; (4) 0.1 ml of biotinylated secondary antibody (IgG) for 8 min; (5) 0.1 ml of avidin-HRPO conjugate (horseradish peroxidase), which binds to the biotin, for 8 min; (6) 0.1 ml of diaminobenzidine (DAB) for 8 min. This chromagen produces a brown precipitate at the sites of the avidin–biotin reaction; (7) 0.1 ml of copper sulphate to enhance the brown precipitate; (8) 0.1 ml of haematoxylin and 0.1 ml of bluing agent containing lithium carbonate to stain the nuclei blue. The slides were then dehydrated through graded alcohols and mounted and fixed with xylene and DPX. Normal bladder tissue controls and breast cancers with *HER2/neu* gene amplification and strong protein overexpression were used as controls in each run.

### Scoring

Representative areas were identified from the H&E sections and corresponding areas were scored using a conventional light microscope. Only membrane staining was scored, with cytoplasmic staining being ignored. A 4-point scale was used: ‘0’ if there was no membrane staining, ‘1’ if there was weak membrane staining in at least 10% of cells, ‘2’ if there was moderate membrane staining in at least 10% of cells, ‘3’ if there was strong membrane staining in at least 10% of cells.

## RESULTS

### Patients

The average age of the patients was 71.3 years (range 43–92) and there were 20 male and five female patients. The average time to progression from preinvasive disease was 20.6 months (range 2–90). There was one postoperative death, eight patients were still alive and 16 (16/25, 64% had died from their disease. The average survival from the time of the diagnosis of muscle-invasive disease to death was 9.9 months (range 1–57). In the majority of cases detrusor muscle invasion was preceded by either a pT1G3 (13 out of 26 cases) or a pTaG2 (10/26 cases) tumour. Tables 1a

**Table 1a:** The HER2/chromosome 17 ratio, HER2 copy number, chromosome 17 copy number, IHC score and time to progression in months for all 26 tumour pairs

**Patient number**	**HER2/chromosome 17 ratio**	**Chromosome 17 copy number**	**HER2 copy number**	**IHC score**	**Time to progression (months)**
1a	1.06	2.75	2.89	‘3’	13
1b	1.25	3.48	4.33	‘3’	
2a	1	1.74	1.73	‘3’	62
2b	1.17	1.93	2.25	‘2’	
3a	1.21	3.86	4.68	‘2’	9
3b	1.16	3.41	4.03	‘1’	
4a	1.13	2.33	2.6	‘3’	3
4b	1.51	3.19	4.97	‘3’	
5a	1.02	1.83	1.87	‘3’	38
5b	1.06	1.81	1.9	‘0’	
6a	1.09	1.98	2.14	‘3’	2
6b	1.5	2.13	3.17	‘3’	
7a	1.09	3.49	3.83	‘3’	3
7b	0.98	2.96	2.93	‘2’	
8a	0.82	3.28	2.61	‘3’	19
8b	1	8.48	8.5	‘3’	
9a	0.95	2.22	2.09	‘2’	32
9b	1.07	2.08	2.21	‘1’	
10a	1.06	2.77	2.9	‘2’	10
10b	0.83	2.51	2	‘0’	
11a	0.92	2.35	2.15	‘3’	4
11b	0.96	2.63	2.49	‘3’	
12a	0.91	2.7	2.47	‘3’	42
12b	0.98	3.15	3.07	‘3’	
13a	0.78	3.26	2.5	‘3’	12
13b	0.88	2.78	2.4	‘3’	
14a	0.96	3.39	3.24	‘3’	17
14b	0.9	3.43	3.08	‘3’	
15a	2.18	2.9	6.33	‘3’	6
15b	2.09	3.01	6.2	‘3’	
16a	1.14	2.43	2.74	‘1’	5
16b	1.48	4.7	7.04	‘0’	
17a	8.07	2.3	18.3	‘3’	26
17b	1.45	2.26	3.17	‘0’	
18a	0.96	1.91	1.82	‘0’	3
18b	2.02	1.62	3.2	‘0’	
19a	1.11	2.26	2.54	‘0’	3
19b	1.06	3.89	4.07	‘0’	
20a	0.98	1.97	1.93	‘0’	90
20b	1.33	3.43	2.58	‘0’	
21a	1.02	1.91	1.94	‘0’	10
21b	0.99	2.01	1.98	‘0’	
22a	1.04	2.13	2.21	‘2’	55
22b	1.06	3.69	3.79	‘3’	
23a	1.05	2.2	2.33	‘3’	6
23b	1.06	2.16	2.29	‘3’	
24a	1.07	3.62	3.8	‘3’	15
24b	0.96	2.98	2.86	‘0’	
25a	0.95	1.84	1.75	‘0’	31
25b	0.69	5.5	3.83	‘0’	

and 1b

**Table 1b:** Stage and grade distribution of the 25 pairs of tumours

	**G3 pT2**	**G2 pT2**	**G3 pT4**	**G2 pTa**	**G3 pT1**	**G1 pTa**	**G1 pT1**	**G2 pT1**
Preinvasive				10	12	1	1	1
Postinvasive	22	1	2					

The table illustrates that most (23/2523/26) of the postinvasive tumours were G3 pT2, and there were 11 pTa and 14 pT1 tumours in the preinvasive group.

give the stages and grades of the tumours, the FISH and IHC results as well as the times to progression.

### Fluorescence *in situ* hybridisation

The average *HER2/neu* copy number for the preinvasive tumours was 3.34 (range 1.73–18.30) and 3.48 for the postinvasive tumours (range 1.90–8.50), and these were not statistically different (*P*=0.086). Overall, 19 out of 25 (76%) of the preinvasive and 23 out of 25 (92%) of the postinvasive tumours had an increased *HER2/neu* copy number. The average chromosome 17 copy number for the preinvasive group was 2.58 (range 1.74–3.68) and 3.16 (range 1.62–8.48) for the postinvasive group, and again these were not statistically different (*P*=0.067). Overall 23 out of 25 (92%) of the preinvasive group and 24 out of 25 (96%) of the postinvasive group were polysomic for chromosome 17. The average *HER2*/chromosome 17 ratio in the preinvasive group was 1.34 and 1.17 in the postinvasive group; these values were not statistically different (*P*=0.31). These results are summarised in Table 2

**Table 2:** Mean HER2 copy number, chromososme 17 copy number and HER2/chromosome 17 copy ratio in the pTa and p T1 tumours

	**Mean *HER2* copy number (range)**	**Mean chromosome 17 copy number (range)**	**Mean *HER2*/chromosome 17 ratio (range)**
pTa	3.34	2.58	1.34
(*n*=11)	(1.7–18.3)	(1.74–3.68)	(082–2.18)
pT1	3.53	3.16	1.17
(*N*=15)	(1.9–8.5)	(1.62–8.48)	(0.91–8.07)

There was no difference in the rates of polysomy 17 (*P*=0.21), *HER2/neu* copy number (*P*=0.34) or *HER2*/chromosome 17 (*P*=0.44) ratio between the pTa and pT1 tumours.

. Two tumours out of both the pre- and postinvasive groups were amplified for *HER2/neu*, representing three patients in total. The stages and grades of these four tumours are given in Table 4.

### Immunohistochemistry

In all, 19 (19 out of 25, 76%), of the preinvasive tumours and 13 (52%) of the postinvasive tumours were ‘positive’ for *HER2/neu* protein overexpression and therefore six (six out of 25, 24%) of the preinvasive and 12 (48%) of the postinvasive tumours were ‘negative’ (Table 3

**Table 3:** Values for the *HER2* immunohistochemistry results

	**‘0’**	**‘1+’**	**‘2+’**	**‘3+’**
Preinvasive	5	1	4	15
Postinvasive	10	2	2	11

The HER2 immunohistochemistry results for the 25 pairs of patients. Both ‘2+’ and ‘3+’ were considered positive and ‘0’ and ‘1+’ were considered negative. In total, 19 out of 25 76% of the preinvasive tumours were positive. The level of protein overexpression was less in the postinvasive tumours (13 out of 26, 50%).

).

## DISCUSSION

Contemporary models of bladder TCC progression suggest that tumours of a higher stage and grade have accumulated more genetic changes (Simon *et al*, 1998). It is thought that genetic changes occur in sequence, such that certain genetic changes are more common in pT2 compared with pTa/T1 tumours (Sauter *et al*, 1998). In the study by Simon *et al* (1998), the overall average number of genetic aberrations in pT1 tumours was 9.8, in comparison with 3.7 in pTa tumours, a statistically significant difference (*P*<0.01). The authors concluded that these two tumour groups are very different, both genetically and in terms of clinical behaviour, with the pT1 tumours more likely to progress to detrusor muscle-invasive disease than the pTa tumours, which did not progress. In the present study, there were 11 pTa and 14p T1 tumours (Tables 1a and 1b), but in contrast to the study by Simon *et al*, all the tumours in this study progressed to detrusor muscle invasion. Table 2 compares the pTa tumours with the pT1 tumours in terms of HER2 copy number, chromosome 17 copy number and protein overexpression, demonstrating that they were not statistically different. This suggests that the subgroup of pTa tumours that are genetically different from pT1 tumours, as suggested by Simon *et al*, are those that are unlikely to progress to pT2 disease. However, in the present study, all the pTa/pT1 tumours progressed to pT2 disease and as such had a completely different clinical course. Hence, in terms of tumour progression, tumour stage and grade (Table 4

**Table 4:** Stage and grade details of the four gene amplified tumours, together with FISH results

**Tumour stage/grade**	**HER2 copy number**	**Chromosome 17 copy number**	**HER2/chromosome 17**
G2 pTa	6.33	2.99	2.18
G3 pT2	6.2	3.01	2.09
G3 pT1	18.3	2.3	8.07
G3 pT2	3.22	1.6	2.01

The tumours with *HER2*/chromosome 17 ratios of 2.18 and 2.09 are from the same patient, suggesting that gene amplification is present before the onset of muscle invasion and persists. The preinvasive G3 pT1 tumour with the highest level of amplification had a postinvasive *HER2*/chromosome 17 ratio of 1.45. The postinvasive G3 pT2 tumour with borderline gene amplification of 2.01 had a preinvasive *HER2*/chromosome 17 ratio of 0.96.

) appear to be less important than the actual genetic changes that a tumour has accumulated. This study suggests that those tumours that progress to pT2 disease have acquired significant HER2/*neu* abnormalities before muscle invasion. Oncogene activation is thought to occur late, and most genetic changes are thought to occur before disease progression (Tsao *et al*, 2000). It therefore appears that in tumours that progress to pT2 disease have already acquired *HER2/neu* abnormalities that occur before the onset of detrusor muscle invasion.

Gene amplification rates were low, being present in 8% of both tumour groups, a value similar to previously published rates of 7 and 9% (Sauter *et al*, 1993; Underwood *et al*, 1995). The results are also similar to those recently published by our group where polysomy *c-myc* and *CCND1* rates were higher than gene amplification rates (Watters *et al*, 2002). Polysomy has been shown to occur independently of tumour polyploidy and is not only chromosome specific but also closely related to tumour progression (Watters *et al*, 2002).

High levels of *HER2/neu* protein overexpression were also observed, with rates of 76 and 52% present in pTa/T1 and pT2 tumours, respectively. These values were not significantly different (*P*=0.06), again suggesting that high level protein overexpression occurs before tumour progression. It has been suggested that either transcriptional or post-transcriptional mechanisms are responsible for the observed difference between protein overexpression and gene amplification. This phenomenon has also been observed in breast cancers. In one study, 22 out of 79 (29%) of breast cancers had *HER2/neu* protein overexpression without *HER2/neu* gene amplification (Farabegoli *et al*, 1999). In a previous study by Sauter *et al* (1993), 89% of bladder tumours with *HER2/neu* protein overexpression did not have gene amplification, results which are similar to the present study. Transcription rates, and hence *HER2/neu* protein expression are controlled by nuclear concentrations of the transcription factors such as GATA-3 and OB2-1 (Shiga *et al*, 1993; Hollywood and Hurst, 1995). Higher levels of such transcription factors, even in the absence of gene amplification, result in increased *HER2/neu* protein overexpression. Stomach cancer cell lines SNU-1 and SNU-16 have similar *HER2/neu* transcription rates with similar mRNA concentrations, but the SNU-1 cells express the *HER2/neu* protein at a higher level than the SNU-16 cells. This is due to preferential translation of the mRNA from the SNU-1 cells (Bae *et al*, 2001).

Anti-*HER2/neu* therapy has been used to treat breast cancers in a clinical setting with encouraging results. Herceptin is a monoclonal antibody directed against the *HER2/neu* protein, which has an antimitotic and antiangiogeneic effect on tumours cells. In breast cancer, response rates have been highest in tumours that have strong protein overexpression (which are also gene amplified). Overall response rates of 50% have been observed (Slamon *et al*, 2001). Furthermore, synergism has been demonstrated between Herceptin and conventional chemotherpeutic agents like cisplatin (Baselga, 2001). The high rates of strong (‘3+’) protein overexpression rates in the pTa/T1 tumours 60%, together with the high rates of polysomy 17 and increased *HER2/neu* copy number suggests that Herceptin might also be of value in the treatment of TCC. In particular, the application of anti-*HER2/neu* therapy to pTa or pT1 tumours that are most likely to progress to pT2 disease, based upon the presence of increased HER2 copy number, polysomy 17 and increased *HER2/neu* protein overexpression might lower the rate of disease progression.
